# Characteristics associated with use of subcutaneous depot medroxyprogesterone acetate (DMPA-SC) in Burkina Faso, Democratic Republic of Congo, and Uganda

**DOI:** 10.1016/j.conx.2021.100055

**Published:** 2021-01-20

**Authors:** Philip Anglewicz, Elizabeth Larson, Pierre Akilimali, Georges Guiella, Patrick Kayembe, Simon P.S. Kibira, Fredrick Makumbi, Scott Radloff

**Affiliations:** a Johns Hopkins Bloomberg School of Public Health, Department of Population, Family and Reproductive Health; b School of Public Health, University of Kinshasa, Kinshasa, Democratic Republic of the Congo; c Institut Supérieur des Sciences de la Population, University of Ouagadougou, Ouagadougou, Burkina Faso; d Makerere University School of Public Health, Kampala, Uganda

## Abstract

**Objectives:**

To what extent is DMPA-SC reaching new users versus encouraging method switching among existing users? Though increasingly-popular, little is known about characteristics of women using DMPA-SC in SSA. We compared characteristics of women using DMPA-SC with those of other modern methods, and identified the extent to which women using DMPA-SC switched from another method or are first-time users of contraception.

**Study design:**

We used data collected by the Performance Monitoring for Action (PMA) Project between 2016 and 2019 from three countries, Burkina Faso, Democratic Republic of Congo, and Uganda. We tabulated characteristics of DMPA-SC, DMPA-IM, implant, and male condom users, and used multivariate analysis to compare characteristics of women using DMPA-SC those of the other three methods. We also examined previous contraceptive method use (if any) among women currently using DMPA-SC.

**Results:**

We found that never-married women were more likely to use male condoms instead of DMPA-SC. Women with two or more children (compared to no children or one child) were more likely to use implants instead of DMPA-SC in both Uganda and DRC. DMPA-SC was the first method used by the majority of current users in Burkina Faso and Uganda. DMPA-SC users who previously used another method generally switched from less effective methods.

**Conclusions:**

Although the characteristics of women using DMPA-SC varied across countries, DMPA-SC appears to be reaching new populations of women instead of inspiring existing modern users to switch to DMPA-SC, and appears to be appealing to first time users of contraception.

**Implications:**

It appears that DMPA-SC appeals to new contraceptive users in sub-Saharan Africa, which implies that DMPA-SC may have the potential to increase modern contraceptive prevalence in sub-Saharan African countries.

## 1 Introduction

Subcutaneous depot medroxyprogesterone acetate (DMPA-SC) (also known by the product name “Sayana®Press”), has the potential to become a prominent modern contraceptive method in sub-Saharan Africa (SSA). Injectables are already the most common modern method in SSA [1], and DMPA-SC has some notable advantages over intramuscular DMPA (DMPA-IM): DMPA-SC can be self-injected or administered by a community health worker (CHW), and is increasingly available at pharmacies or drug shops, thereby potentially reaching a larger number of women than DMPA-IM [[2], [3], [4], [5], [6]]. In some settings, women who used both methods preferred DMPA-SC due to fewer side effects, faster administration, and less pain [4]; and family planning service providers preferred the method as well [7]. In addition, self-injection of DMPA-SC was found to be more cost-effective than DMPA-IM [8,9]. Given these attributes, it's not surprising that the prevalence of DMPA-SC has increased in recent years [10].

The rapid introduction of DMPA-SC to contraceptive markets in SSA has implications for the use of DMPA-IM, other modern methods, and the trajectory of modern contraceptive use in the region. There is hope that DMPA-SC will be appealing and accessible to women in SSA who have not previously used modern contraception, which may then increase modern contraceptive prevalence in the region [2,11,12]. Evidence suggests that the addition of a new contraceptive method often attracts new contraceptive users and increases overall contraceptive prevalence [13]. But the extent to which DMPA-SC users have switched from another modern method or are first-time contraceptive users at the population level is not known. Among women currently using DMPA-SC, what methods did they use previously, if any?

Little is known about characteristics of women using DMPA-SC and how they compare with those of other modern methods in SSA, particularly DMPA-IM. Most studies of DMPA-SC users are not population-based, instead involving sub-populations like adolescents [11] or family planning clients of community health workers or service delivery facilities [[14], [15], [16], [17]]. The few population-based studies have examined a limited set of DMPA-SC user characteristics, and have seldom compared characteristics of DMPA-SC users with women using other prominent contraceptive methods [6,18,19]. One population-based study recently compared characteristics of DMPA-SC with users all other modern methods combined and non-users [7], but how characteristics of women using DMPA-SC compare with DMPA-IM and other specific modern methods is not known. A comparison of characteristics of DMPA-SC users to users of other common modern methods (particularly DMPA-IM) provides insight into whether DMPA-SC is indeed reaching new populations, or simply reaching the same population as other modern methods.

In this research, we used population-based data from Burkina Faso, the Democratic Republic of Congo (DRC), and Uganda to (1) compare characteristics of women using DMPA-SC with those of the most common other non-permanent modern methods in each country, and (2) measure the percentage of DMPA-SC users who switched from other methods (and if so, from which methods), or were new users of modern contraception.

## 2 Materials and methods

Few population-based datasets include measures of DMPA-SC in SSA countries. Demographic and Health Surveys (DHS) recently added questions about DMPA-SC to the round 8 survey instrument [20], but results have not yet been published.

For this analysis, we used data from the Performance Monitoring for Action Project (PMA) (previously called “Performance, Monitoring and Accountability 2020”). Since 2013, PMA has collected population representative data on key family planning indicators in eleven geographies in Africa and Asia. In all countries, PMA used multi-stage stratified cluster design to draw a probability sample of households and females of childbearing age. Datasets can be obtained through the PMA website at www.pmadata.org; more information on the study is provided in Zimmerman et al. 2017 [21].

We used PMA data from Burkina Faso, Uganda, and the DRC. We selected these countries because they have multiple rounds measuring DMPA-SC use, and each has achieved DMPA-SC prevalence of greater than 1% among all women in the most recent PMA survey. Data from the first two countries are representative at the national level, while PMA in DRC operates in two provinces, Kinshasa and Kongo Central, selecting representative samples at this subnational level. The specific rounds of PMA data used are from 2016, 2017, 2018, and 2019 for Burkina Faso; 2016, 2017 and 2018 for DRC; and 2017 and 2018 for Uganda. PMA received ethical approval from institutional review boards in each country and Johns Hopkins Bloomberg School of Public Health.

### 2.1 Measures

PMA surveys measured contraceptive use (including DMPA-SC) with a series of questions, starting with “Are you or your partner currently doing something or using any method to delay or avoid getting pregnant?” If the woman reported using contraception, the PMA survey then asked her to name the method or methods she was using. If the woman reported using an injectable, the survey asked “Was the injection administered via syringe or small needle?” and respondents were shown an image of both DMPA-SC and DMPA-IM so she could provide an accurate distinction.

We compared characteristics of DMPA-SC users with three other prominent modern methods in each country. Because DMPA-SC has similar attributes and is often compared with DMPA-IM (e.g., [4,6]), we included DMPA-IM users in our analysis for all three countries. The other two methods were the most common non-permanent modern methods among all women in each country, which were the same in all three settings: implants and male condoms.

We measured method switching among DMPA-SC users through the following question: “Right before you started using [current/most recent method], were you doing something or using any method to delay or avoid getting pregnant?” Respondents were then asked for the penultimate method used (these questions were included only in Burkina 2018 and 2019 and 2018 DRC survey instruments). We tabulated this for current DMPA-SC users to identify previous methods used (if any). We also tabulate responses to the question “Which method did you first use to delay or avoid getting pregnant?” among current DMPA-SC users to identify the percentage for whom DMPA-SC was the first method used.

The characteristics in our analysis were selected as those which are commonly associated with contraceptive use and method type. We focused on three categories of measures: (1) sociodemographic characteristics, (2) fertility preferences, and (3) exposure to family planning messages. The sociodemographic measures are age, number of lifetime births, marital status, level of education, household wealth, and urban/rural residence. Household wealth was measured using a constructed wealth index based on ownership of 25 household durable assets, house and roof material, livestock ownership and water source. We also included family planning programming, measured as whether the woman was exposed to a family planning message via radio, television, or a magazine. Finally, we included two family planning-related measures: fertility preferences (want another birth), and an index of the number of FP methods known (ranging from 0 to 13).

### 2.2 Analytic methods

In our analysis, we first presented the (weighted) percentages of characteristics of interest for women using DMPA-SC, DMPA-IM, implants, and male condoms. We used bivariate chi-squared tests with design-based F statistics to identify statistically significant differences in these characteristics between DMPA-SC users and users of each other method. For measures with multiple categories (e.g., age), we compared all categories as a group between women using DMPA-SC and those of the other three methods.

To examine whether and how users of DMPA-SC are different from women using each of these other modern methods, we conducted multivariable regressions. The purpose of these regressions is to identify characteristics associated with DMPA-SC use compared to other methods, while controlling for other measures that are associated with contraceptive use and method choice. We used multinomial logistic regression in which the binary dependent variable was DMPA-SC users (with value of “0”), compared to users of each of the other three contraceptive methods. Independent variables included were age, which was separated into five year age intervals from 15 to 49 years; number of births (divided into categories of 0–1, 2–5, 6 or more), highest level of educational attainment (none, primary, secondary, tertiary), marital status (currently married/living together, divorced/ widowed, never-married), wealth quintile, the FP program exposure measures, fertility preferences (want another child, do not want another child, don't know/infertile) and number of contraceptive methods known (0–13). We presented adjusted relative risk ratios (aRRR) and 95% confidence intervals (95% CI) for all regression results. We accounted for the study design features and non-response by using survey weights in our analysis.

Finally, we examined previous method use among women currently using DMPA-SC by tabulating (1) the penultimate contraceptive method among women currently using DMPA-SC (in Burkina Faso and DRC only), and (2) the first contraceptive method used among women currently using DMPA-SC in each country, which also included the percentage of women for whom DMPA-SC was their first contraceptive method.

## 3 Results

Table 1 shows characteristics of women using DMPA-SC, DMPA-IM, implants, and male condoms; and results of chi-squared statistical tests comparing characteristics of women using the latter three methods to those using DMPA-SC. Overall, our bivariate results show that, compared to users of DMPA-SC, condom users were typically younger, better educated, and were unmarried. In all settings, condom users were more likely to be younger, never married, and to have 0–1 children than DMPA-SC users. Condom users were also better educated and more likely to see an advertisement about FP in a magazine in Burkina Faso and Uganda. Condom users were aware of more methods in Burkina Faso but fewer in Uganda and DRC. It appears that implant users have significantly higher parity than DMPA-SC users in all settings. There were few statistically significant differences between users of DMPA-SC and DMPA-IM or implants.

**Table 1:** Characteristics of women using DMPA-SC, compared with DMPA-IM Implants, and Male Condoms in Burkina Faso, DRC, and Uganda.

	Burkina Faso				DRC				Uganda			
	DMPA-SC	DMPA-	Implants	Male condom	DMPA-	DMPA-	Implants	Male	DMPA-	DMPA-	Implants	Male
		IM			SC	IM		Condom	SC	IM		Condom
n =	253	427	1107	434	75	207	444	673	167	868	482	276
**Age category**												
15–19	6.7%	9.7%	**8.9%**	**25.8%**	7.0%	6.0%	6.2%	**19.1%**	5.2%	8.7%	4.9%	**27.1%**
20–24	22.3%	17.3%	**19.6%**	**32.6%**	26.7%	17.2%	21.7%	**29.0%**	22.3%	25.5%	26.4%	**25.1%**
25–29	28.6%	24.6%	**20.3%**	**17.4%**	16.4%	19.1%	17.1%	**21.9%**	31.1%	24.5%	27.5%	**18.0%**
30–34	19.0%	21.8%	**20.2%**	**9.8%**	16.4%	20.8%	24.8%	**14.2%**	18.4%	19.2%	20.8%	**12.6%**
35–39	15.0%	16.1%	**14.9%**	**9.0%**	12.4%	15.6%	14.9%	**9.0%**	14.3%	13.1%	12.3%	**10.6%**
40–44	6.7%	8.1%	**11.7%**	**3.2%**	10.0%	16.0%	11.4%	**4.5%**	5.7%	6.2%	6.5%	**4.1%**
45–49	1.9%	2.5%	**4.4%**	**2.1%**	11.0%	5.3%	4.0%	**2.4%**	3.2%	2.8%	1.7%	**2.5%**
p-value		0.47	**0.04**	**< 0.01**		0.39	0.28	**0.02**		0.67	0.80	**< 0.01**

**Number of children**												
0–1	16.3%	19.2%	**17.9%**	**28.8%**	30.3%	17.9%	**14.3%**	**38.4%**	24.0%	22.1%	**13.3%**	**44.9%**
2–5	65.5%	57.6%	**52.3%**	**22.6%**	50.9%	54.8%	**60.3%**	**32.8%**	55.2%	56.9%	**64.4%**	**29.5%**
6 +	18.1%	23.2%	**29.8%**	**48.6%**	18.9%	27.4%	**25.4%**	**28.8%**	20.8%	21.1%	**22.3%**	**25.6%**
p-value		0.17	**0.01**	**< 0.01**		0.13	**0.03**	**0.02**		0.90	**0.04**	**< 0.01**

**Fertility preferences**												
More children	84.1%	78.4%	**76.8%**	89.6%	61.9%	52.9%	57.6%	**82.3%**	72.5%	67.9%	64.2%	82.6%
No more children	15.4%	19.3%	**22.4%**	10.2%	30.6%	37.8%	33.3%	**15.8%**	26.8%	31.8%	35.7%	17.2%
Don't know/infertile	0.5%	2.3%	**0.9%**	0.2%	7.5%	9.3%	9.1%	**1.9%**	0.7%	0.3%	0.2%	0.2%
p-value		0.12	**< 0.01**	0.14		0.53	0.71	**< 0.01**		0.56	0.10	0.18

**Education**												
None	62.7%	62.6%	65.5%	**16.2%**	4.8%	9.5%	5.1%	6.6%	5.0%	5.5%	8.5%	**3.6%**
Primary	21.6%	22.1%	18.6%	**13.6%**	26.9%	28.7%	31.6%	17.6%	40.6%	34.9%	38.1%	**24.7%**
Secondary	14.8%	14.7%	14.9%	**55.1%**	67.5%	61.1%	61.5%	67.6%	49.6%	53.0%	45.8%	**50.0%**
Tertiary or higher	0.9%	0.6%	1.0%	**15.1%**	0.9%	0.7%	1.8%	8.3%	5.0%	6.7%	7.7%	**21.8%**
p-value		0.96	0.72	**< 0.01**		0.63	0.81	0.16		0.66	0.44	**0.01**

**Marital Status**												
Married	93.6%	92.0%	89.0%	**34.7%**	73.9%	75.2%	73.8%	**48.5%**	86.0%	78.7%	86.1%	**46.7%**
Divorced/widowed	2.4%	2.1%	4.5%	**3.8%**	13.2%	11.0%	10.9%	**3.7%**	9.7%	10.6%	11.0%	**10.9%**
Never-married	4.0%	5.9%	6.6%	**61.5%**	12.9%	13.8%	15.3%	**47.9%**	4.4%	10.8%	2.9%	**42.5%**
p-value		0.57	0.05	**< 0.01**		0.88	0.83	**< 0.01**		0.15	0.69	**< 0.01**

**Wealth quintile**												
1 (lowest)	19.6%	21.0%	22.7%	**6.6%**	4.8%	11.2%	16.5%	12.9%	17.7%	12.4%	18.0%	9.8%
2	20.9%	22.4%	21.6%	**6.7%**	15.0%	21.8%	19.4%	16.6%	17.3%	18.5%	20.4%	14.1%
3	16.8%	22.5%	21.8%	**8.3%**	16.0%	16.5%	20.4%	21.4%	20.8%	24.1%	21.2%	17.4%
4	22.3%	14.7%	16.4%	**14.1%**	22.9%	26.2%	17.1%	20.1%	26.9%	23.0%	17.1%	18.2%
5 (highest)	20.4%	19.5%	17.5%	**64.3%**	41.3%	24.4%	26.6%	29.0%	17.3%	21.9%	23.2%	40.5%
p-value		0.29	0.29	**< 0.01**		0.25	0.05	0.33		0.64	0.47	0.08
**Heard FP on radio**	69.7%	64.9%	67.4%	65.3%	43.7%	31.1%	34.7%	35.6%	76.2%	84.7%	78.3%	83.4%
p-value		0.33	0.52	0.35		0.09	0.08	0.17		0.13	0.73	0.23
**Saw FP on TV**	37.4%	33.3%	**30.2%**	**60.4%**	33.9%	33.6%	50.7%	37.0%	30.9%	29.8%	25.7%	48.4%
p-value		0.45	**0.04**	**< 0.01**		0.98	0.25	0.83		0.88	0.49	0.11
**Saw FP in magazine**	10.9%	11.4%	10.6%	**29.0%**	6.8%	11.9%	7.8%	12.6%	16.7%	22.0%	16.1%	**33.3%**
p-value		0.88	0.93	**< 0.01**		0.46	0.82	0.25		0.24	0.88	**0.04**
**Mean number of FP methods known**	6.9	**7.5**	7.0	**8.5**	7.4	7.1	7.7	**6.4**	9.2	9.0	9.0	**8.5**
p-value		**0.02**	0.97	**< 0.01**		0.45	0.34	**< 0.01**		0.18	0.30	**0.01**
**Urban residence**	18.5%	20.3%	21.1%	**68.9%**	-	-	-	-	20.3%	20.5%	17.9%	32.8%
p-value		0.65	0.42	**< 0.01**						0.98	0.81	0.41

**Notes:** Bold font indicates statistically significant differences between characteristics of DMPA-SC users and other methods, by measure group (at p < 0.05); p-values provided are from bivariate analysis were chi-squared tests with design-based F statistics; urban residence was not measured in the sampling frame for DRC, so it is not included in the analysis; the data source for this analysis was the Performance Monitoring for Action (PMA) Project, 2016–2019.

Results of multivariate multinomial logistic regressions are shown in Table 2, Table 3, Table 4, separately for each country, and presented by adjusted relative risk ratios (aRRR) and 95% confidence intervals (CIs). In Burkina Faso (Table 2), we found that knowledge of one additional FP method was associated with a greater aRRR of using DMPA-IM instead of DMPA-SC (aRRR 1.17; 95% CI 1.06–1.30). We did not find any statistically significant differences in characteristics between women using implants and DMPA-SC. Women who had six or more children, had secondary or tertiary or high education, were divorced/widowed and never-married, lived in an urban area, and knew more FP methods all had significantly higher risk of using male condoms instead of DMPA-SC.

**Table 2:** Characteristics of women using DMPA-IM, Implants, and Male Condom compared with DMPA-SC, in Burkina Faso.

	DMPA-IM		Implants		Male condom	
	aRRR	95% CI	aRRR	95% CI	aRRR	95% CI
**Age category**						
15–19 (reference)	1.00		1.00		1.00	
20–24	0.66	0.28, 1.52	0.84	0.41, 1.75	0.76	0.35, 1.66
25–29	0.79	0.30, 2.06	0.74	0.33, 1.65	0.55	0.22, 1.39
30–34	0.93	0.35, 2.49	0.98	0.40, 2.40	0.63	0.23, 1.74
35–39	0.67	0.23, 1.90	0.74	0.32, 1.75	0.70	0.24, 2.03
40–44	0.58	0.17, 1.99	1.14	0.38, 3.47	0.45	0.14, 1.47
45–49	0.53	0.11, 2.63	1.39	0.31, 6.28	0.85	0.16, 4.45

**Number of children**						
0–1 (reference)	1.00		1.00		1.00	
2–5	0.65	0.38, 1.11	0.73	0.44, 1.20	1.10	0.59, 2.04
6 +	0.71	0.35, 1.46	1.20	0.60, 2.40	**4.78**	**2.22, 10.25**

**Fertility preferences**						
More children (reference)	1.00		1.00		1.00	
No more children	1.65	0.88, 3.12	1.12	0.69, 1.83	1.05	0.52, 2.14
Don't know/infertile	7.09	0.74, 67.49	1.36	0.16, 11.9	0.70	0.06, 8.86

**Education**						
None (reference)	1.00		1.00		1.00	
Primary	0.82	0.47, 1.43	0.82	0.51, 1.33	1.22	0.67, 2.24
Secondary	0.73	0.41, 1.30	0.86	0.50, 1.47	**2.13**	**1.05, 4.33**
Tertiary or higher	0.32	0.07, 1.40	0.77	0.27, 2.20	**4.42**	**1.37, 14.29**

**Marital Status**						
Married (reference)	1.00		1.00		1.00	
Divorced/widowed	0.77	0.25, 2.41	1.59	0.72, 3.50	**3.04**	**1.12, 8.21**
Never-married	1.39	0.58, 3.34	1.41	0.69, 2.87	**10.68**	**4.54, 25.12**

**Wealth quintile**						
1 (lowest, reference)	1.00		1.00		1.00	
2	1.10	0.67, 1.81	0.93	0.55, 1.57	0.89	0.32, 2.51
3	1.29	0.61, 2.74	1.27	0.65, 2.47	1.43	0.50, 4.13
4	0.59	0.29, 1.20	0.70	0.37, 1.32	1.47	0.53, 4.06
5 (highest)	0.73	0.28, 1.90	0.74	0.32, 1.70	3.40	0.98, 11.75
**Heard FP on radio**	0.80	0.49, 1.32	1.02	0.69, 1.50	0.91	0.51, 1.64
**Saw FP on TV**	0.78	0.40, 1.51	0.71	0.47, 1.09	0.86	0.45, 1.63
**Saw FP in magazine**	0.98	0.52, 1.84	1.03	0.51, 2.10	1.00	0.50, 2,00
**Number of FP methods known**	**1.17**	**1.06, 1.30**	1.03	0.94, 1.14	**1.24**	**1.09, 1.41**
**Urban residence**	1.52	0.75, 3.07	1.62	0.93, 2.82	**2.47**	**1.05, 5.81**
N =	2221					

**Notes**: Results above are from multinomial logistic regressions. Instances where the odds ratios in 95% CIs do not cross 1 are indicated in bold font; aRRR = adjusted relative risk ratios (adjusted); 95% CIs = 95% confidence intervals; the data source for this analysis was the Performance Monitoring for Action (PMA) Project, 2016–2019. We included all measures above as variables in these regression models.

**Table 3:** Characteristics of women using DMPA-IM, Implants, and Male Condom compared with DMPA-SC users, in DRC.

	DMPA-IM		Implants		Male condom	
	aRRR	95% CI	aRRR	95% CI	aRRR	95% CI
**Age category**						
15–19 (reference)	1.00		1.00		1.00	
20–24	0.91	0.30, 2.74	0.86	0.35, 2.12	0.68	0.27, 1.72
25–29	1.26	0.31, 5.17	0.71	0.31, 1.66	0.95	0.25, 3.65
30–34	1.31	0.26, 6.70	1.05	0.28, 3.94	0.93	0.17, 5.09
35–39	0.94	0.26, 3.43	0.60	0.23, 1.58	0.73	0.21, 2.59
40–44	0.92	0.21, 3.96	0.48	0.15, 1.57	0.51	0.08, 3.17
45–49	0.30	0.08, 1.17	**0.14**	**0.04, 0.47**	0.42	0.08, 2.19

**Number of children**						
0–1 (reference)	1.00		1.00		1.00	
2–5	1.67	0.61, 4.60	**3.04**	**1.40, 6.57**	1.39	0.67, 2.86
6 +	1.76	0.56, 5.53	**3.47**	**1.17, 10.30**	2.35	0.91, 6.07

**Fertility preferences**						
More children (reference)	1.00		1.00		1.00	
No more children	1.74	0.83, 3.65	1.49	0.89, 2.51	0.59	0.33, 1.07
Don't know/infertile	2.99	0.89, 10.05	**3.12**	**1.50, 6.50**	0.51	0.17, 1.53

**Education**						
None (reference)	1.00		1.00		1.00	
Primary	0.65	0.11, 3.92	1.08	0.21, 5.46	0.54	0.09, 3.45
Secondary	0.67	0.15, 2.97	0.88	0.21, 3.69	0.99	0.21, 4.75
Tertiary or higher	0.43	0.02, 7.77	2.50	0.21, 29.69	10.42	0.79, 137.62

**Marital Status**						
Married (reference)	1.00		1.00		1.00	
Divorced/widowed	1.05	0.47, 2.35	1.03	0.43, 2.48	0.43	0.17, 1.13
Never-married	1.31	0.44, 3.89	1.16	0.31, 4.31	**3.60**	**1.20, 10.77**

**Wealth quintile**						
1 (lowest, reference)	1.00		1.00		1.00	
2	0.67	0.18, 2.49	0.35	0.09, 1.34	0.41	0.13, 1.28
3	0.48	0.10, 2.19	0.43	0.11, 1.66	0.60	0.18, 1.92
4	0.56	0.08, 4.04	0.28	0.05, 1.46	0.35	0.08, 1.47
5 (highest)	0.29	0.05, 1.65	0.22	0.04, 1.11	**0.25**	**0.07, 0.93**
**Heard FP on radio**	0.55	0.24, 1.24	**0.51**	**0.29, 0.89**	0.77	0.35, 1.69
**Saw FP on TV**	1.54	0.50, 4.76	2.94	0.92, 9.42	1.19	0.33, 4.24
**Saw FP in magazine**	4.98	0.92, 26.90	1.91	0.57, 6.40	**3.95**	**1.03, 15.16**
**Number of FP methods known**	0.90	0.76, 1.06	1.02	0.88, 1.19	**0.77**	**0.66, 0.90**
N =	1399					

**Notes**: Results above are from multinomial logistic regressions. Instances where the odds ratios in 95% CIs do not cross 1 are indicated in bold font; aRRR = adjusted relative risk ratios (adjusted); 95% CIs = 95% confidence intervals; the data source for this analysis was the Performance Monitoring for Action (PMA) Project, 2016–2019. We included all measures above as variables in these regression models.

**Table 4:** Characteristics of women using DMPA-IM, Implants, and Male Condom compared with DMPA-SC, in Uganda.

	DMPA-IM		Implants		Male condom	
	aRRR	95% CI	aRRR	95% CI	aRRR	95% CI
**Age category**						
15–19 (reference)	1.00		1.00		1.00	
20–24	0.56	0.19, 1.63	0.74	0.24, 2.26	**0.25**	**0.09, 0.67**
25–29	**0.37**	**0.14, 0.96**	0.43	0.15, 1.24	**0.20**	**0.07, 0.57**
30–34	0.44	0.16, 1.21	0.42	0.16, 1.12	**0.26**	**0.08, 0.81**
35–39	0.40	0.11, 1.50	**0.25**	**0.07, 0.87**	0.43	0.10, 1.75
40–44	0.44	0.12, 1.60	0.29	0.07, 1.18	0.34	0.07, 1.61
45–49	0.29	0.05, 1.66	**0.12**	**0.03, 0.51**	0.24	0.03, 2.07

**Number of children**						
0–1 (reference)	1.00		1.00		1.00	
2–5	1.60	0.95, 2.69	**2.66**	**1.42, 4.97**	0.88	0.46, 1.66
6 +	1.38	0.64, 2.97	**2.81**	**1.17, 6.74**	1.16	0.48, 2.84

**Fertility preferences**						
More children (reference)	1.00		1.00		1.00	
No more children	1.53	0.85, 2.76	**1.88**	**1.11, 3.16**	1.00	0.43, 2.33
Don't know/infertile	0.42	0.04, 3.97	0.24	0.02, 3.26	0.33	0.01, 8.09

**Education**						
None (reference)	1.00		1.00		1.00	
Primary	0.62	0.25, 1.58	0.46	0.19, 1.10	0.87	0.20, 3.74
Secondary	0.78	0.27, 2.24	0.53	0.18, 1.56	1.02	0.21, 4.99
Tertiary or higher	1.14	0.30, 4.38	1.16	0.29, 4.64	4.55	0.75, 27.66

**Marital Status**						
Married (reference)	1.00		1.00		1.00	
Divorced/widowed	1.33	0.61, 2.91	1.40	0.58, 3.36	2.49	0.94, 6.64
Never-married	2.74	0.91, 8.28	0.76	0.26, 2.26	**10.70**	**2.34, 48.86**

**Wealth quintile**						
1 (lowest, reference)	1.00		1.00		1.00	
2	1.36	0.65, 2.82	1.16	0.51, 2.65	1.09	0.41, 2.87
3	1.54	0.62, 3.85	1.11	0.42, 2.96	1.26	0.44, 3.59
4	1.17	0.47, 2.91	0.81	0.36, 1.82	0.91	0.29, 2.83
5 (highest)	2.04	0.67, 6.20	2.13	0.65, 6.91	3.17	0.95, 10.54
**Heard FP on radio**	1.87	0.87, 4.02	1.29	0.57, 2.92	1.80	0.79, 4.07
**Saw FP on TV**	0.62	0.37, 1.06	0.62	0.35, 1.12	0.79	0.40, 1.56
**Saw FP in magazine**	1.56	0.93, 2.61	1.21	0.68, 2.15	1.61	0.80, 3.24
**Number of FP methods known**	0.89	0.71, 1.11	0.90	0.70, 1.14	0.78	0.61, 1.00
**Urban residence**	0.96	0.28, 3.29	0.85	0.23, 3.21	0.96	0.20, 4.71
N =	1793					

**Notes**: Results above are from multinomial logistic regressions. Instances where the odds ratios in 95% CIs do not cross 1 are indicated in bold font; aRRR = adjusted relative risk ratios (adjusted); 95% CIs = 95% confidence intervals; the data source for this analysis was the Performance Monitoring for Action (PMA) Project, 2016–2019. We included all measures above as variables in these regression models.

For women in the DRC (Table 3), women who had two to five (aRRR 3.04; 95% CI 1.40–6.57) or six or more children (aRRR 3.47; 95% CI 1.17–10.30), and who didn't know if they wanted more children or were infertile (aRRR 3.12; 95% CI 1.50–6.50) had significantly greater aRRR of using implants instead of DMPA-SC. Women aged 45 to 49 and heard about FP on the radio had significantly lower relative risk of using implants compared to DMPA-SC. Finally, we found that women who were never-married or saw an FP advertisement in a magazine had significantly greater relative risk of using male condoms compared to DMPA-SC; and women who were in the highest wealth quintile and knew more FP methods had significantly lower relative risk of using male condoms instead of DMPA-SC.

Turning to Uganda (Table 4), women who were between 25 and 29 years old had significantly lower relative risk of using DMPA-IM compared to DMPA-SC (aRRR 0.37; 95% CI 0.14–0.96). Women aged 35 to 39 and 45 to 49 had significantly lower relative risk of using implants compared to DMPA-SC; and those with between two and five children, and six or more children, and wanted no more children had significantly higher relative risk of using implants instead of DMPA-SC. For male condom, we found that women aged 20 to 34 had significantly lower relative risk of using male condom (compared to women aged 15 to 19) instead of DMPA-SC; and women who were never-married had significantly higher relative risk (aRRR 10.70; 95% CI 2.34–48.86) of using male condoms instead of DMPA-SC.

Finally, in Table 5 we show results for previous method use (both first method and penultimate method) among women currently using DMPA-SC. Starting with the penultimate method, DMPA-IM was the penultimate method for 13.7% of current DMPA-SC users in Burkina Faso and 11.3% in DRC. The remainder were slightly more likely to have switched to DMPA-SC from a less effective than from a more effective method, particularly in DRC, where 40% of current DMPA-SC users were previously using a less effective method. Turning to first method used, in Uganda, DMPA-SC was the first method for just over half of DMPA-SC users (52.1%), compared to 41.3% in DRC, and 58.0% in Burkina Faso. DMPA-IM was the first method for 25.1% of current users in Uganda, 3.8% in DRC, and 14.6% in Burkina Faso.

**Table 5:** Previous contraceptive method use among women currently using DMPA-SC in Burkina Faso, DRC, and Uganda.

	Burkina Faso				DRC				Uganda	
	Penultimate Method		First Method Used		Penultimate Method		First Method Used		First Method Used	
	n	%	n	%	n	%	n	%	n	%
**More effective method**	**28**	**13.7**	**36**	**12.5**	**0**	**0.0**	**2**	**1.9**	**10**	**6.0**
**Equally effective method**	**28**	**13.7**	**42**	**14.6**	**6**	**11.3**	**4**	**3.8**	**42**	**25.1**
**Less effective method**	**26**	**13.3**	**43**	**14.8**	**21**	**40.4**	**55**	**52.9**	**28**	**16.8**
**DMPA-SC first**	**121**	**59.4**	**167**	**58.0**	**25**	**48.3**	**43**	**41.3**	**87**	**52.1**
Implant	26	12.9	36	12.5	0	0.0	2	1.9	9	5.4
IUD	2	0.8	0	0.0	0	0.0	0	0.0	1	0.6
DMPA-IM	28	13.7	42	14.6	6	11.3	4	3.8	42	25.1
Pill	19	9.2	34	11.8	13	26.0	21	20.2	7	4.2
Emergency	0	0.2	0	0.0	1	2.2	0	0.0	5	3.0
LAM	0	0.0	0	0.0	0	0.0	1	1.0	0	0.0
Male condom	3	1.3	5	1.7	2	3.8	5	4.8	7	4.2
Rhythm	2	1.2	3	1.0	3	6.3	15	14.4	3	1.8
Withdrawal	1	0.7	1	0.3	0	0.0	4	3.8	1	0.6
Other	1	0.7	0	0.0	1	2.2	9	8.7	5	3.0
Total	204	100.0	288	100.0	51	100.0	104	100.0	167	100.0

**Notes**: Penultimate method was only asked in the final two rounds in Burkina Faso and final round in DRC; respondents who did not report a first method were dropped (2 in Burkina Faso, 5 in DRC, 2 in Uganda). IUDs and implants were considered more effective methods than DMPA-SC; DMPA-IM was considered equally effective; pills, emergency contraception, LAM, male condom, rhythm, withdrawal, and other methods were considered less effective than DMPA-SC; the data source for this analysis was the Performance Monitoring for Action (PMA) Project, 2016–2019.

## 4 Discussion

Our goal in this research is to examine whether DMPA-SC is reaching a new population of contraceptive users. To do so, we used data from three countries in SSA with growing prevalence of DMPA-SC use [7], and compared characteristics of women using DMPA-SC with those using other common non-permanent modern methods, and examined the extent to which current DMPA-SC users switched from another method- and if so, which method they previously used.

Overall, we find that characteristics of women using DMPA-SC generally differed from those using other common modern contraceptive methods. Some differences in characteristics are consistent across countries. In all three countries, never-married women were more likely to use male condoms instead of DMPA-SC, which is consistent with research elsewhere showing that never-married women were less likely to use DMPA-SC than all other modern methods combined [7]. This may be explained by the fact that condoms are generally not used in marriage in SSA [22]. We also find that, compared to women aged 15 to 19, those aged 45 to 49 were less likely to use implants instead of DMPA-SC in Uganda and DRC, which could be the result of DMPA-SC programs targeted to younger women in these settings [23]. Overall, however, the relationship between age and DMPA-SC use (compared to other methods) was not consistent across settings, which conforms to results of DMPA-SC expansion in Nigeria, which also reached a high percentage of new users of contraception but lower percentages of women under 25 [6]. Finally, women with two or more children (compared to none or one) were more likely to use implants instead of DMPA-SC in both Uganda and DRC. Other differences in characteristics were not consistent across countries, which likely reflects differing approaches to the introduction of DMPA-SC across countries, as described in Stout et al. 2018 [18].

Since they are considered comparable methods, it is notable that there were relatively few differences in characteristics between users of DMPA-SC and DMPA-IM in our multivariate analysis. This lack of differences in characteristics is consistent with previous research from Uganda, but not in Burkina Faso, where prior research found differences between DMPA-SC and DMPA-IM users in age, parity, education, wealth [17].

In addition, we find that DMPA-SC was the first method for the majority of current users in Burkina Faso and Uganda, and for close to half of current users in DRC. Less than 15% of current DMPA-SC users switched from DMPA-IM in Burkina Faso and DRC. We also found that many current DMPA-SC users in DRC switched from a less effective method, which might reflect the fact women often initiate contraceptive use with short-acting and less effective methods [25]. Overall, we find evidence for the speculation that DMPA-SC may be appealing for first-time users of contraception [24], again supporting our conclusion that DMPA-SC appears to be reaching new populations.

This research has some notable limitations. First, we do not have information on penultimate method used in Uganda, so we cannot measure method switching there. Also, penultimate method was measured only in 2018 in DRC, leading to a relatively small sample size. In addition, among DMPA-SC users who previously used another method, we do not have information on the timing of previous method use. Therefore, we do not know if the woman switched from the previous method immediately, or if time elapsed between method use (for example, a pregnancy and birth could have occurred in between methods). Third, the number of DMPA-SC users is still relatively small in some settings, which has implications for our analysis.

Although this research focuses on individual characteristics, use of DMPA-SC is also likely related to features of the community, particularly the supply-side features of DMPA-SC, starting with access to DMPA-SC. In the three countries here, access to DMPA-SC has increased in recent years: in Burkina Faso, DMPA-SC was available in 85% of public facilities nationally by the end of 2016 [18]. Despite this expansion in access, however, stockouts of DMPA-SC were not uncommon in Burkina Faso and Uganda in 2016 [24]. Another relevant community-level measure of DMPA-SC is the mode of provision: DMPA-SC is also provided by community health workers, and can be self-administered, but the availability of these modes varies within and across countries [17,18]. Since the characteristics of DMPA-SC users differ by how and where the method was received [19], this might also partly explain our results here. Finally, the quality of counseling provided is another supply-side feature that impacts use of DMPA-SC and other methods, and may explain our results here [26]. We intend to examine patterns of supply-side features of DMPA-SC (compared to other methods) in the future.

Overall, our results have positive implications for the role of DMPA-SC in the increase of modern contraceptive use in SSA. For many users, DMPA-SC was their first contraceptive method. Many others switched from a less effective contraceptive method in DRC, but less so in Burkina Faso and Uganda. Similarly, women using DMPA-SC were generally different from those of other prominent modern methods in each country. This all implies that DMPA-SC is indeed reaching new populations of women instead of inspiring existing users to switch methods, and that DMPA-SC could therefore increase the overall modern contraceptive prevalence with expanded access.

## Funding

This article was developed under grants INV-009639 GI and INV-009643 awarded to Johns Hopkins Bloomberg School of Public Health by the Bill & Melinda Gates Foundation. The funding source was not involved in this research.

## Declaration of competing interests

The authors declare that they have no known competing financial interests or personal relationships that could have appeared to influence the work reported in this paper.
